# Advantageous/Unfavorable Effect of Quercetin on the Membranes of SK-N-SH Neuroblastoma Cells

**DOI:** 10.3390/molecules26164945

**Published:** 2021-08-16

**Authors:** Barbara Kreczmer, Barbara Dyba, Anna Barbasz, Elżbieta Rudolphi-Szydło

**Affiliations:** Institute of Biology, Pedagogical University, Podchorążych 2, 30-084 Kraków, Poland; barbara.dyba@up.krakow.pl (B.D.); anna.barbasz@up.krakow.pl (A.B.); elzbieta.rudolphi-szydlo@up.krakow.pl (E.R.-S.)

**Keywords:** quercetin, neuroblastoma cells, cell membrane, Langmuir monolayer, oxidative stress

## Abstract

Quercetin is a polyphenolic compound, the effects of which raise scientists’ doubts. The results of many experiments show that it has anticancer, antiinflammatory, and antioxidant properties, while other studies indicate its pro-oxidative and cytotoxic action. This compound can react with reactive oxygen species, and due to its chemical properties, it can be found in the hydrophobic-hydrophilic area of cells. These features of quercetin indicate that its action in cells will be associated with the modification of membranes and its participation in maintaining the redox balance. Therefore, this study distinguishes these two mechanisms and determines whether they are important for cell function. We check: (1) Whether the selected concentrations of quercetin are cytotoxic and destructive for SK-N-SH cell membranes (MTT, LDH, MDA tests) in situations with and without the applied oxidative stress; (2) what is the level of changes in the structural/mechanical properties of the lipid part of the membranes of these cells due to the presence of polyphenol molecules; and (3) whether the antioxidative action of quercetin protects the membrane against its modification. Our results show that changes in the stiffness/elasticity of the lipid part of the membrane constitute the decisive mechanism of action of quercetin, potentially influencing cellular processes whose initial stages are associated with membranes (e.g., reception of signals from the environment, transport).

## 1. Introduction

Quercetin (3,3′,4′,5,7-pentahydroxyflavone) is a bioactive polyphenol occurring in plants [1,2,3], and is the most abundant flavonoid in the human diet [4].

It exhibits a wide range of properties—among antiinflammatory [5] and immunomodulatory [6]. Literature data indicate that it may be a potential anticancer factor [7,8]. It owes its action to the ability to modify the course of intracellular signaling pathways [9,10] and to antioxidant properties that have been demonstrated, among others, on PC12 cells [11,12] and human neuronal SH-SY5Y cells [13].

This compound is an antiproliferative agent, inducing programmed cell death in a number of tumor lines, for example, in HL-60 and NB-4 cells from acute myelogenous leukemia [14,15] and K-562-chronic myelogenous leukemia [16]. It was also shown that quercetin inhibits the propagation of various types of cancers, such as in the lung, liver, breast, and colon [17,18,19,20]. Vijayababu et al. [21] found that it induces apoptosis of prostatic carcinoma cells (PC-3), as well as inhibits invasion, migration, and signaling molecules involved in cell survival and proliferation of this type of cells [22].

However, the action of quercetin is not clear. There are also data indicating high cytotoxicity of this compound [23,24,25]. Research shows that quercetin, depending on the concentration, location in the cell, and the source of free radicals, may show a pro-oxidative effect. High doses generated oxidative stress in human lymphocytes [26].

Such dualistic action of quercetin may be related to the fact that as an antioxidant it may become an oxidation product. The result of this reaction is the formation of a semiquinone radical which oxidizes to quercetin quinone [2]. This compound can react with thiol groups becoming toxic to cells [26].

Another possibility for beneficial/unfavorable action on cells is the effect of this compound on membranes. Due to the chemical nature, the presence of aromatic rings in the molecule, it can locate itself in hydrophobic cell compartments, thus changing its physicochemical properties [27,28]. Mechanical and structural properties of the lipid part of membranes are very important for the course of all processes related to membranes, i.e., transport, signaling, and stimuli conduction. These processes are mainly carried out by proteins anchored in membranes. Changes in the stiffness or flexibility of the membrane may block the possibility of adopting the appropriate conformation of individual protein domains, which may consequently disrupt or inhibit the process carried out by a given protein. It is, therefore, likely that changing the membrane composition by introducing even a small amount of quercetin between lipid molecules can significantly change their mobility.

In view of existing literature ambiguity as to the effects of quercetin on cells, this research evaluates the anti or pro-oxidative action of quercetin and to check the influence of this compound on the functioning of cell membranes, including the determination of the structural modification of the lipid part of these membranes. This goal was achieved by examining the effect of different concentrations of quercetin on the survival of SK-N-SH cells and the level of damage to their membranes. The potential antioxidant activity of this compound was checked by initiating oxidative stress to cells after prior incubation with quercetin. The action at the membrane level was tested in model systems giving unambiguous information about: (1) The structural modification of the lipid monolayer of composition reflecting the cell membrane (SK-N-SH) caused by the presence of various amounts of quercetin, and (2) the protective abilities of the tested flavonoid against peroxidation of unsaturated fatty acids in the lipids that build the cell membrane (SK-N-SH).

## 2. Results

### 2.1. Native Cell Membranes

The effect of quercetin on human SK-N-SH cells was investigated using the MTT assay, which determines mitochondrial activity.

**Figure 1:**
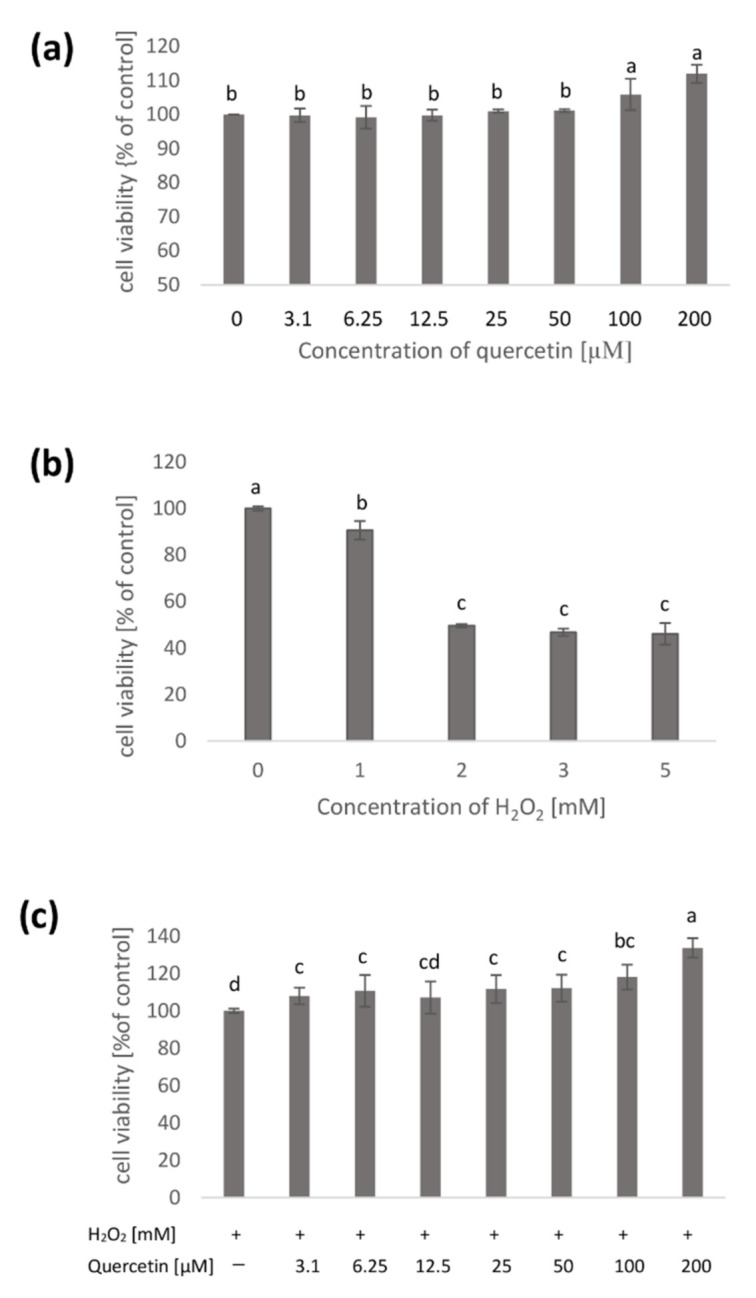
The cell viability was determined by MTT assay. SK-N-SH cells were exposed for 24 h to various concentrations of quercetin (3.1–200 µM). The percentage of cell viability was referred to the untreated control cells (**a**); cells contacted for 3 h with various concentrations of H_2_O_2_ (mM) (**b**), SK-N-SH cells preincubated for 24 h with different concentrations of quercetin and then contacted for 3 h with H_2_O_2_. The percentage of cell viability was related to cells treated only with H_2_O_2_ (**c**). Values represent mean ± SD of three to six independent experiments. Different letters indicate significant differences (*p* ≤ 0.05).

SK-N-SH were exposed for 24 h to various concentrations of quercetin (3.1, 6.25, 12.5, 25, 50, 100, and 200 µM). Cell treatment with lower concentrations of quercetin did not show any significant effect. In contrast, the addition of quercetin at a concentration of 200 µM caused a slight increase in the number of viable cells (11% compared to control) (Figure 1a).

The study also investigated the effect of varying concentrations of hydrogen peroxide (1–5 mM) on the cells after 3 h contact. It was observed that the lowest tested concentration caused a slight decrease in cell viability (by about 10% compared to the control), while higher concentrations were toxic to SK-N-SH cells. In the presence of H_2_O_2_ at 3 and 5 mM concentrations in the culture medium, a significant (of approximately 54%) decrease in cell viability was noted compared to cells not treated with H_2_O_2_ (Figure 1b).

To determine the protective effect of quercetin against H_2_O_2_ toxicity, cells were treated with various concentrations of quercetin for 24 h followed by 3 h contact of the sample with hydrogen peroxide of 3 and 5 mM. It was observed that quercetin of 3.1–50 µM concentrations caused a slight, about 9%, increase in the number of viable cells, as compared to cells treated with H_2_O_2_ alone. In contrast, for SK-N-SH cells preincubated in a medium containing higher quercetin concentrations (100 and 200 µM) and then contacted with H_2_O_2_, cell viability increased by approximately 18 and 33% (Figure 1c).

The next stage of the work was to check to what extent quercetin and H_2_O_2_ affect the membranes of the tested cells. For this purpose, the lactate dehydrogenase (LDH) test was used to assess the level of cell membrane damage, and the malondialdehyde (MDA) level was measured to evaluate the degree of lipid peroxidation. The SK-N-SH cells were treated for 24 h with selected concentrations of quercetin (6.25; 25; 100; 200 µM), and then the hydrogen peroxide (3–5 mM) was added for another 3 h.

As shown in Table 1 the presence of quercetin in the medium used for 24 h treatment of SK-N-SH cells did not cause any significant change in LDH release.

**Figure 2:**
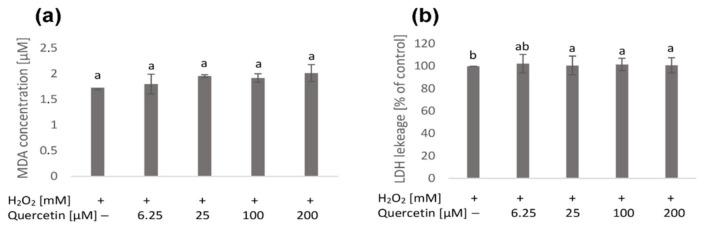
The effect of quercetin on SK-N-SH cells exposed to H_2_O_2_ was measured by the degree of lipid peroxidation (concentration of MDA) (**a**) and LDH release (**b**). The cells were pretreated for 24 h with selected quercetin concentrations (6.25; 25; 100; 200 µM), followed by 3 h contact with H_2_O_2_ (3–5 mM). Percentage of LDH leakage was referred to values obtained for cells treated with H_2_O_2_ only (**b**). Values represent mean ± SD of three to six independent experiments. Different letters indicate significant differences (*p* ≤ 0.05).

Pretreatment with a quercetin concentration of 6.25 µM did not influence MDA content, while the higher doses of quercetin (25; 100 and 200 µM) induced an increase in lipid peroxidation by about 12% (Figure 2a).

Treatment of the cells with both quercetin and hydrogen peroxide did not affect LDH release in comparison to cells treated with hydrogen peroxide alone (Figure 2b).

### 2.2. Model Membranes

#### 2.2.1. The Influence of Quercetin on the Mechanical Properties of Membranes

**Figure 3:**
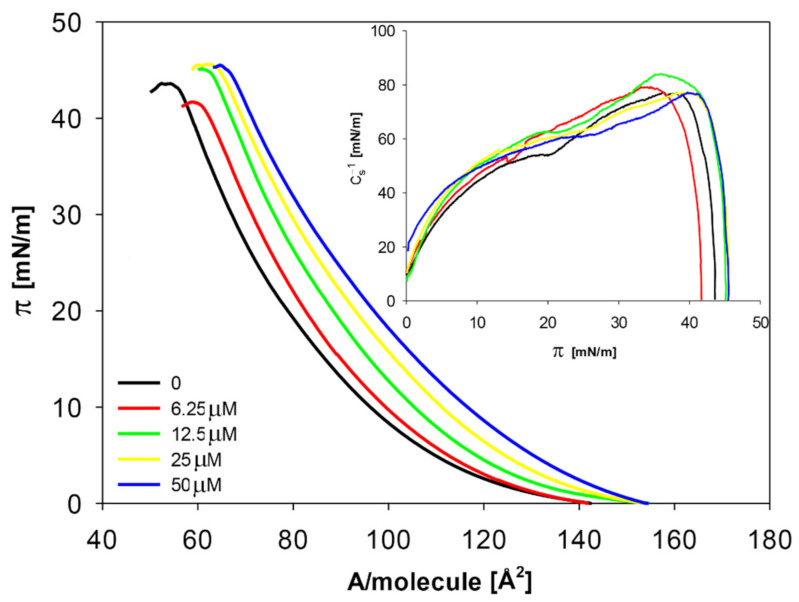
Surface pressure isotherms and corresponding compressibility modulus (inset) of the lipid model of neuroblastoma layers spread on the subphase contained the indicated concentrations of quercetin dissolved in buffer.

To test the effect of quercetin on the membranes of neuroblastoma cells, lipid monolayers (of a composition reflecting the lipid part of the membranes of the SK-N-SH cell line) were formed. Monolayers were made on pure buffer pH 7.4 (control) and on a buffer containing quercetin at concentrations of 6.25, 12.5, 25, and 50 µM. For such monolayers, the dependencies of surface pressure (π) on the area per single molecule in the monolayer (A) were measured (Figure 3).

The course of (π-A) isotherms obtained for the lipid mixture mimicking composition characteristic for the neuroblastoma membrane is characterized by a monotonic course, without noticeable phase transitions. The presence of quercetin clearly modifies this relationship, causing a shift in the curves towards higher A values. This tendency is directly proportional to the concentration of quercetin in the buffer solution. The dependence of the static C_s_^−1^ compression modulus on π was calculated based on the isotherms (inset in Figure 3).

Based on the obtained isotherms, the values of physicochemical parameters characterizing the tested monolayers were determined (Table 2).

With increasing quercetin amount, A_lim_ (minimal area per molecule in the densely packed layer) increased proportionally to its concentration. For the highest level of quercetin, this increase was as large as 17.5% compared to the control. The remaining parameters, i.e., π_coll_ and C_s_^−1^ (surface pressure at the collapse point of the layer and static compression modulus), were less sensitive to the quercetin content in the subphase. The changes in πcoll ranged from 3–5%, and for C_s_^−1^ from 0.2 to 8.8% compared to the control.

#### 2.2.2. Antioxidant Activity of Quercetin

**Figure 4:**
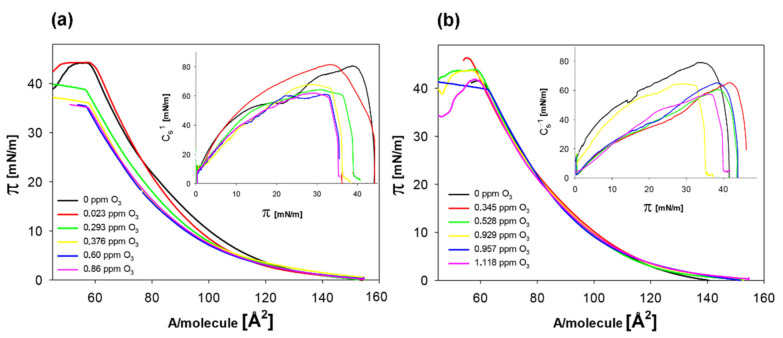
Surface pressure isotherms and corresponding compressibility modulus (inset) of lipid model of neuroblastoma layers spread on the subphase contained the indicated concentrations of ozone dissolved in buffer. Isoterms: (**a**) in the absence of antioxidants; (**b**) in the presence of quercetin (6.25 µM) added to the ozone-containing buffer.

To test the effectiveness of quercetin in protecting membranes against oxidative stress, experiments were performed in which the lipid models of the membrane were exposed to ozone in the absence (Figure 4a) and presence of quercetin at a concentration of 6.25 µM (Figure 4b).

Under the influence of ozone, the course of both the dependencies, i.e., π = f(A) and C_s_^−1^ = f(π) changes. The isotherms obtained for monolayer spread on the ozone-containing subphase were characterized by a smaller slope and a shift towards lower A values. Moreover, C_s_^−1^ values decreased under the influence of ozone. These changes were greatest at an ozone concentration of about 0.4 ppm. A further increase of ozone concentration did not increase the changes in monolayers characteristics (Figure 4a inset).

When quercetin at a concentration of 6.25 µM was introduced into the subphase, ozone did not significantly affect the course of the π(A) isotherm; however, the values of the compression modulus changed in ozone presence similarly as in the case of quercetin absence (Figure 4b inset).

The numerical values of the parameters characterizing the monolayers determined based on the obtained isotherms are presented in Table 3.

In the presence of ozone, a slight reduction in the surface area per molecule (A_lim_) in densely packed monolayer is observed. The maximum reduction of this parameter is 3.2% compared to the control (monolayer on the pure buffer). The C_s_^−1^ parameter is also reduced, reaching plateau values at ozone concentrations above 0.6 ppm O_3_. The maximum reduction of this parameter reaches 24% in relation to the control.

When quercetin at a concentration of 6.25 µM is present in the subphase, A_lim_ values (1–2%) decreases slightly, while the reduction in the C_s_^−1^ value is about 27%.

**Figure 5:**
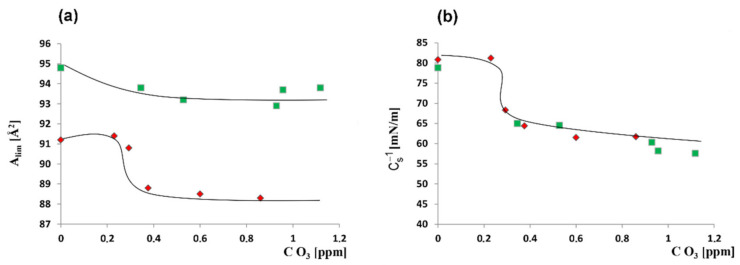
The dependence of the area per molecule on ozone concentration (**a**) and dependence of compression modulus on ozone concentration (**b**) for neuroblastoma membrane on subphase: Without quercetin-red points; with quercetin-green points. Lines do not present any physical model, were mathematically fitted, and were drawn for the convenience of tracking parameter changes.

The nature of the A_lim_ changes in the presence of ozone and of ozone in combination with quercetin have different courses. At an ozone concentration of about 0.3 ppm, in the absence of the quercetin, the A_lim_ decreases abruptly to about 88.3 Å^2^. It then reaches the minimum plateau value giving characteristic S-shape dependence (Figure 5a, red points). In the presence of quercetin, there is a slight smooth monotonic decrease of A_lim_, reaching higher ozone concentration a plateau of about 93.5 Å^2^ (Figure 5a, green points).

The maximal layer compression modulus values change similarly with ozone concentration for both: In the absence and the presence of quercetin (Figure 5b).

## 3. Discussion

The mechanism of action of quercetin has been intensively studied for many years, but the results of experiments are often contradictory and indicate that in some circumstances, this compound is beneficial, and in others, unfavorable for cells. The analysis of the available studies shows that the effect of this compound on cells is related to two aspects, i.e., its interaction with membranes and antioxidant activity. However, in cells, it is not possible to distinguish these two modes of quercetin action and the assessment of the suitability of this compound as a potential substance, e.g., having antitumor activity. For this reason, the experiments have been planned in such a way as to assess in a qualitative and quantitative manner the influence of this compound on the mechanical properties of membranes, as well as its antioxidant activity in the model system, which eliminates the effects of the activity of other metabolic pathways.

Based on the performed experiments, it was confirmed that even at the lowest concentrations (6.25 µM), quercetin modifies (as indicated by the increase in A_lim_ value) the model monolayers, mimicking the lipid part of the membrane of neuroblastoma cells. It points to a possibility of the interaction of quercetin with the membranes of these cells when reaching the brain, even at low concentrations. This is of particular importance for nerve cells whose membranes contain relatively large amounts of lipids.

The approximate lipid-protein ratio for nerve cells is 80:20%, and for example, for the erythrocyte membrane, this ratio is 50:50% [29]. Such a level of lipids in the membrane of nerve cells is linked to the role of these cells in transmitting electrical signals. At the same time, even subtle changes in physicochemical properties, such as flexibility and stiffness, of the cell membrane, can significantly disturb this function.

The action potential is transferred along the membrane of the nerve cell thanks to the work of ion channels, and then, thanks to the action of ion pumps, the resting potential is restored. The proper functioning of these proteins (in particular, determined by their shape) is closely dependent on the lipid environment. Moreover, changes in the mechanical properties of the membrane may disturb the operation of channels whose gating mechanism depends precisely on the mechanical properties of the membrane.

These theses are confirmed by studies that showed that the effect of quercetin on the cell membrane is related, inter alia, to the transport of Ca^2+^ ions and/or Ca^2+^ metabolism [30,31]. Thus, the introduction of quercetin molecules into the environment of the membrane (especially of nerve cells) may have significant consequences for their functioning.

To check whether quercetin action determined in model systems plays a beneficial or unfavorable role in cells, the results obtained for model membranes were compared with the effect of this compound on whole neuroblastoma cells. For SK-N-SH cells, the MTT test was done, giving the information about cell viability of cells which in turn is directly proportional to mitochondrial dehydrogenase enzymes.

The obtained results showed that the presence of quercetin at concentrations of 100 and 200 µM increased the number of viable cells. Similar results were obtained by Bao et al. [32], investigating the effect of quercetin on PC-12 cells. The LDH leakage into the extracellular environment, which gives information about the disruption of the cell membrane, was also examined at the same quercetin levels. The obtained results demonstrated that the membranes of the tested cells were not damaged under such conditions. Boots et al. [2], studying the effects of quercetin on lung epithelial cells, observed similar results. Therefore, it can be concluded that at these concentrations, quercetin has a positive effect on cells, increasing their viability.

The second important aspect of the mechanism of quercetin action is related to its antioxidant properties. To assess the associated effect of the studied polyphenol, experiments were carried out in which it was checked whether quercetin plays a protective role in cells exposed to oxidative stress. Oxidative stress was generated by introducing H_2_O_2_ into the cell environment. As demonstrated in this study, hydrogen peroxide causes significant cell mortality, expressed as damages to the mitochondria.

When SK-N-SH cells were cultured for 3 h in a medium containing 3 and 5 mM H_2_O_2_, the cell viability decreased to approximately 44% of that of untreated cells (MTT assay). The studies showed that 24 h pretreatment of cells with quercetin causes an increase in the number of living cells in relation to cells treated with H_2_O_2_. Suematsu et al. [13] reported similar effects.

We also measured the level of MDA, which is a biomarker of oxidative stress as an end product of lipid peroxidation [33]. Cells pretreatment with high quercetin concentrations, followed by the addition of hydrogen peroxide, resulted in a slight increase of MDA content in comparison to cells exposed to H_2_O_2_ alone. This may be because quercetin (at higher concentrations) can also undergo oxydo-reductive activation and be a subject of redox cycling, generating intracellularly reactive oxygen species [34].

There were no significant changes in membrane destruction (as indicated by unchanged LDH level) of cells treated with quercetin and H_2_O_2_ compared to the case, when cells were exposed to oxidative stress only. Therefore, it can be concluded that despite an increase in lipid peroxidation (at high quercetin concentrations), this was not significant enough to destroy the membranes and disrupt their continuity.

The observations obtained for the native system were confronted with those received for a model system where it is possible to control exactly both quercetin concentration and stress level. It was checked how the lipid part of the neuroblastoma membrane is resistant to oxidative stress. Stress conditions were created by introducing ozone into the buffer, where in the water environment ozone undergoes decomposition reactions forming oxidative radicals, among others hydroxyl radicals and superoxide anion radicals [35]. The obtained mixture of reactive oxygen species corresponds to those naturally occurring in cells, due to the redox status disturbance imbalance [36,37,38].

Under the influence of stress, the shape of isotherms changed, indicating a significant decrease of the area per single molecule in the densely packed monolayer. Such changes took place due to the oxidation of unsaturated bonds in the fatty acid chains. This effect increased with the increase in ozone concentration to about 0.3 ppm, and then this isotherm parameter remained at the same level despite the increase in ROS. This means that at ozone concentration of about 0.3 ppm, all unsaturated fatty acids present in the monolayer were oxidized (in the neuroblastoma model, the total amount of unsaturated fatty acids was 52%).

Oxidized monolayers have substantial stiffness, as represented by the C_s_^−1^ value (the higher the value of this parameter, the larger is the stiffness of the layer). In the presence of quercetin, even at a low concentration (6.25 µM), the modifications of the membrane exposed to ozone, as visualized by changes of the A_lim_ value, were significantly reduced (Figure 5a), which proves the antioxidant activity of this compound. It is worth noting, however, that although quercetin scavenged the radicals, protecting the unsaturated fatty acids against oxidation, it did not reverse the changes in membrane stiffness (Figure 5b).

Thus, it has been shown that quercetin exhibits an antioxidant action, but its mere presence between lipid molecules influences (determines) the parameters of the layer, i.e., despite the protection of fatty acid chains against oxidation, the membrane stiffness decreased significantly (C_s_^−1^ value about 25% lower compared to the control).

These results indicate that it is the membrane change that is the main factor of the cellular effects resulting from the presence of quercetin. However, the consequences of this change (modification of signaling, transport) vary depending on the number of polyphenol molecules, the initial composition of the membrane, and the function of the cell. This explains why, in different circumstances and in different studies, quercetin shows a protective effect, while in other quite the opposite.

In a situation of high oxidative stress, the antioxidant effect helps the cell by protecting its components against damage. However, if the membranes are significantly modified by locating this compound in their environment resulting in the distribution of the signaling or transport pathways by changed membrane stiffness, this may lead to unfavorable effects. It should also be remembered that many processes in a cell take place simultaneously, both those related to membranes and those that generate oxidative stress. The effect that can be observed and measured by determining the level of specific indicators is always total. Hence, there are situations in which the effect of quercetin is perceived as favorable and unfavorable.

## 4. Materials and Methods

### 4.1. Materials

The composition of the model of the lipid part of the neuroblastoma cells membrane was established based on the work by the authors of [39,40,41]. Phospholipids: 1-oleoyl-2-palmitoyl-sn-glycero-3-phosphocholine; 1-hexacosanoyl-d4-2-hydroxy-*sn*-glycero-3-phosphocholine; 1,2-dioleoyl-sn-glycero-3-phosphocholine; L-α-phosphatidylethanolamine (Brain); sphingomyelin (Brain)–(Avanti Polar Lipids Inc., Alabaster, AL, USA). Cholesterol was purchased (Sigma-Aldrich, Darmstadt, Germany). Phospholipid to cholesterol ratio was 79% to 21%. The ratio of saturated to unsaturated fatty acids was 48% to 52%.

The solvents (chloroform, ethanol) of chemical purity–POCh (Gliwice, Poland); freshly deionized water produced by HLP 5 Hydrolab (Straszyn, Poland). The culture medium, serum, and antibiotics were purchased from CytoGen GmbH. The chemical reagents used in the experiments were obtained from Sigma Aldrich.

### 4.2. Cell Culture

Human neuroblastoma cell line SK-N-SH (The European Collection of Authenticated Cell Cultures (ECACC) was cultured in 10% fetal bovine serum (FBS) in Dulbecco’s Modified Eagle Medium (DMEM), supplemented with 0.01% penicillin-streptomycin at 37 °C in a humidified atmosphere, containing 5% CO_2_.

### 4.3. Measurement of Cell Viability (MTT Assay)

Cells were seeded in a 96-well plate at a density 1 × 10^4^ cells per well in a volume of 0.1 mL. The cells were treated for 24 h with various concentrations of quercetin (3.75–200 µM). After this time, 3 mM and 5 mM hydrogen peroxide was added for 3 h. Due to the lack of differences between the effects of H_2_O_2_ at 3 and 5 µM concentrations, for statistical studies, they were treated as the same treatments and described as 3–5 µM H_2_O_2_ treatment.

After then 50 µL MTT (3-[4,5-dimethylthiazol-2-yl]-2,5 diphenyl tetrazolium bromide) solution (sterile stock solution of 5 mg/mL) was added to cells and incubated for 2 h at 37 °C in a humidified 5% CO_2_ atmosphere. Then, 0.4 mL of dimethyl sulfoxide (DMSO) was added to each well and kept for 10 min. After centrifugation, the optical density of the supernatant was measured at 570 nm using a microliter plate reader (BioTek Instruments, VT, USA).

### 4.4. Membrane Damage Assay (LDH Assay)

The lactate dehydrogenase (LDH) assay was used as a marker of cell membrane entirety.

Cells in the amount of 1 × 10^4^ cells per well were seeded in a 96-well plate and incubated in the presence of quercetin (3.75–200 µM) for 24 h; Then, 3 mM and 5 mM H_2_O_2_ was added for 3 h. To the tubes containing 0.5 mL of 0.75 mM sodium pyruvate and 10 µL NADH (140 µM) (heated at 37 °C for 10 min), 150 µL of supernatant was added, and incubated for 30 min at 37 °C. Then 0.5 mL of 2,4-dinitrophenylhydrazine was added to the sample, and after 1 h the absorbance was measured at 450 nm.

### 4.5. Determination of Lipid Peroxidation (MDA Concentration)

Membrane lipid peroxidation was estimated by thiobarbituric acid (TBA) reaction with malondialdehyde (MDA). Cells were seeded in 46-well plates containing quercetin and H_2_O_2_ (in concentrations and times described above) in the amount of 1 × 10^4^ cells per well in a volume of 0.25 mL. After treatment, the samples were collected and centrifuged (1000× *g*, 5 min). To the pellets, 0.5 mL of 0.5% TCA was added, vortexed for 1 min, and lysed through sonicating for 5 min. After centrifugation at 10,000× *g* for 10 min, 0.4 mL of supernatant was added to the 1.25 mL 20% TCA with 0.5% TBA and heated in dry thermoblock (100 °C) for 30 min. After cooling, the absorbance was measured at 532 nm using the molar extinction coefficient of MDA equal to 155 M^−1^ cm^−1^.

### 4.6. Model Membranes

Buffer Ozonation. Phosphate buffer (0.01 M, pH 7.4) was saturated with ozone produced by ozone generator FM 500 (Grekos, Poland). The concentration of ozone in a buffer was determined according to Bader and Hoigne [42].

Surface Pressure Isotherms. Langmuir trough (KSV, Finland) was used for surface pressure isotherm registration at a constant compression rate corresponding to 5 mm/min barrier speed. Lipid monolayer was formed by spreading a defined amount of lipid chloroform solution at 1 mg/mL concentration on the subphase composed of aqueous 0.01 M phosphate buffer, pH 7.4 with or without quercetin, and with or without ozone. Surface tensions were measured with a Pt-Wilhelmy plate. Experiments were performed at 25 °C ± 1 °C.

### 4.7. Statistical Analysis

Three to six independent analyses were performed for each tested variant and averaged (±SD). The significant differences compared to the controls were estimated exploiting the SAS ANOVA procedure. The statistical analysis was performed by Duncan’s multiple range test, taking *p* < 0.05. Statistical tests were carried out using STATISTICA 13.3 (Cracow, Poland).

## Figures and Tables

**Table 1:** LDH leakage in SK-N-SH cells treated for 24 h with various concentrations of quercetin (3.1–200 µM). Dehydrogenase lactate release in control untreated cells was defined as 100%. Values represent mean ± SD of three to six independent experiments. Different letters indicate significant differences (*p* ≤ 0.05).

Concentration of Qercetin [µM]	LDH Leakage [% of Control]
0	100 ^a^
3.1	96.98 ± 0.86 ^c^
6.25	99.27 ± 1.02 ^a^
12.5	100.59 ± 1.53 ^a^
25	98.43 ± 0.50 ^b^
50	100.23 ± 2.38 ^a^
100	99.27 ± 0.67 ^a^
200	101.80 ± 0.50 ^a^

**Table 2:** Surface parameters of monolayers (A_lim_–the limiting area per one molecule; π_coll_–collapse pressure; C_s_^−1^–maximal values of compression modulus) calculated for monolayers of the model of neuroblastoma membrane. Data represent mean from three experiments ± SD. Different letters indicate significant differences (*p* ≤ 0.05).

Concentration of Quercetin [μM]	A_lim_ [Å^2^]	π_coll_ [mN/m]	C_s_^−1^_max_ [mN/m]
0	56.4 ± 0.1 ^a^	43.5 ± 0.2 ^b^	76.9 ± 0.2 ^d^
6.25	60.7 ± 0.2 ^b^	42.3 ± 0.3 ^a^	78.8 ± 0.2 ^b^
12.5	62.7 ± 0.1 ^c^	44.8 ± 0.3 ^c^	83.7 ± 0.2 ^a^
25	64.3 ± 0.3 ^d^	45.6 ± 0.2 ^d^	77.1 ± 0.2 ^c^
50	66.3 ± 0.2 ^e^	45.1 ± 0.2 ^e^	76.9 ± 0.2 ^d^

**Table 3:** Surface parameters of monolayers A_lim_ and C_s_^−1^ calculated for monolayers of the model of neuroblastoma membrane spread on the subphase contained the indicated concentrations of ozone dissolved in the buffer in the absence and in the presence of quercetin (6.25 µM). Data represent mean from three experiments ± SD. Different letters indicate significant differences (*p* ≤ 0.05).

O_3_ [ppm]	A_lim_ [Å^2^]	C_s_^−1^_max_ [mN/m]
0	91.2 ± 0.2 ^a^	80.8 ± 0.5 ^a^
0.023	91.4 ± 0.3 ^a^	81.2 ± 0.6 ^a^
0.293	90.8 ± 0.3 ^b^	68.3 ± 0.7 ^b^
0.376	88.8 ± 0.5 ^c^	64.4 ± 0.9 ^c^
0.60	88.5 ± 0.4 ^c^	61.5 ± 1.2 ^d^
0.86	88.3 ± 0.6 ^c^	61.7 ± 1.1 ^d^
+ 6.25 mM quercetin		
0	94.8 ± 0.4 ^a^	78.8 ± 1.2 ^a^
0.345	93.8 ± 0.5 ^b^	65.0 ± 1.4 ^b^
0.528	93.2 ± 0.4 ^b^	64.5 ± 1.3 ^b^
0.929	93.0 ± 0.6 ^b^	60.3 ± 1.1 ^c^
0.957	93.7 ± 0.5 ^b^	58.2 ± 0.9 ^d^
1.118	93.8 ± 0.4 ^b^	57.6 ± 0.8 ^d^

## Data Availability

Data is contained within the article.
